# Latent profiles of resilience associated with problem behaviors among Chinese preschool left-behind children

**DOI:** 10.3389/fpsyg.2026.1801900

**Published:** 2026-10-08

**Authors:** Tianqi Qiao, Haocai Hong, Pingzhi Ye, Jiaxin Xiang, Xinxin Wang

**Affiliations:** 1 School of Education, Guangzhou University, Guangzhou, China; 2 Guangzhou Preschool Teachers College, School of Preschool Education, Guangzhou, China

**Keywords:** latent profile analysis, left-behind children, preschool, problem behaviors, resilience

## Abstract

**Aim:**

This study used latent profile analysis (LPA) to investigate the characteristics of resilience among preschool left-behind children in China. We examined the relationship between resilience and problem behaviors in these children and found that the underlying categories of resilience were related to sociodemographics.

**Design and method:**

A total of 940 preschool left-behind children from kindergartens in the Zhejiang and Shandong provinces were selected as subjects for this cross-sectional study. We used the Personal Information Form, the Devereux Early Childhood Assessment for Preschoolers (Second Edition), and the Social Skills Improvement System-Rating Scales for measurements.

**Results:**

The four resilience profiles for preschool left-behind children showed the best model fit, including the high-resilience group (17.5% of the sample), medium-resilience group (34.5% of the sample), low-resilience group (37.7% of the sample), and risk group (10.3% of the sample). A one-way analysis of variance showed that there were significant differences in problem behaviors among the four groups of children. A multinomial logistic regression analysis showed that child sex, age, parental education, and annual family income had significant predictive effects on the membership of the profiles.

**Conclusion:**

Preschool left-behind children generally lack resilience, which is related to their problem behaviors. Therefore, the results of this study can assist in the development of resilience-focused interventions to promote better psychosocial adjustment among preschool left-behind children.

## 1 Introduction

With the advancement of China’s urbanization, the number of young and middle-aged rural laborers migrating to cities is increasing, and the number of left-behind children in rural areas is also increasing (Lyu et al., 2024). In recent decades, China’s economy has developed rapidly, but the distribution of public resources, such as education, healthcare, and employment, is still unbalanced between urban and rural areas, particularly for left-behind children in rural areas (Jin et al., 2020; Lyu et al., 2024). From the perspective of Bronfenbrenner’s ecological systems theory, a child’s development is profoundly influenced by the interaction between their immediate environment (microsystem) and broader societal structures (macrosystem). For left-behind children, the disruption of their family microsystem due to parental migration creates a developmental void (Bronfenbrenner and Morris, 2006). In this study, left-behind children are operationally defined as those whose parents (one or both) had migrated to other cities for work for at least 6 months, leaving the children in their home communities (typically under the care of one parent, grandparents, or other relatives). These children experience a prolonged lack of parental care, which affects their ability to establish secure attachments, receive emotional support, develop self-regulation, or acquire social skills. According to attachment theory, the absence of primary caregivers during critical periods can hinder the formation of secure internal working models, leading to long-term socioemotional difficulties (Bowlby, 1969). When they remain in this situation for a long time, they tend to develop a variety of externalizing problem behaviors, such as bullying, hyperactivity, and aggressive behaviors (Yu et al., 2022; Zhang et al., 2019; Zhang H. et al., 2021). Additionally, they may become more prone to psychological problems such as autism, social isolation, heightened sensitivity, and paranoia (Luo et al., 2019). The emergence of numerous problem behaviors can seriously hinder children’s cognitive development and is not conducive to children’s physical and mental health development. Due to the particularity of their living environment and the large population of left-behind children, their problem behaviors have attracted wide attention (Dong et al., 2019; Zhou et al., 2026).

At present, the majority of studies on problem behaviors of left-behind children focus on middle-aged and older children, with relatively few studies on left-behind children in preschool (Dong et al., 2019; Jin et al., 2020; Wang et al., 2019; Yu et al., 2022; Zhang et al., 2019). Studies have shown that the younger the children are and the longer they are separated from their parents, the greater their risk of emotional and behavioral problems (Adhikari et al., 2014; Liu et al., 2009). Without early detection and treatment of emotional and behavioral problems in preschool children, these problems may become more severe in adulthood and may even have a series of adverse consequences, such as crime, substance abuse, mental health problems, and poor relationships (Fergusson et al., 2005). Considering that there are 68 million left-behind children in China, more than 40% of whom are younger than 5 years (Luo et al., 2019), it is crucial that we pay more attention to the internalizing and externalizing problems faced by preschool left-behind children.

### 1.1 Resilience

Even in the face of similar disadvantages, only some left-behind children exhibit internalizing and externalizing problem behaviors, whereas other children manage to grow up healthily and transition smoothly from adolescence to adulthood (van Breda, 2017). This difference can be explained by “resilience.” Resilience, which originated from studies of children who adapted well despite adversity, refers to the ability to adapt positively in the face of adversity, which can change over time (Masten et al., 1999). Research has shown that resilience is essential for the development of disadvantaged children. High levels of resilience enable children to overcome adversity, such as poverty, disasters, and abuse (Livingston et al., 2025; Olick, 2016), return to baseline quickly (Edward et al., 2009), and grow up healthily. Besides, studies have shown that children’s resilience levels can predict both internalizing and externalizing problem behaviors (Calkins et al., 2007; Rich et al., 2024). Children and adolescents with high levels of resilience are less likely to report mental health problems and behavioral disorders than those with lower resilience levels (Cao et al., 2024; Rich et al., 2024; Yu et al., 2011). As individuals with low resilience levels are more likely to collapse in the face of difficulties (van Breda, 2017), they have higher levels of problem behaviors.

### 1.2 Latent profile analysis

Although many studies have been conducted using the total score of the resilience scale, the complexity of resilience dimensions means that the general total score does not capture specific pattern information, which hinders a deeper understanding of the nature of resilience. To solve this problem, latent profile analysis (LPA) provides an effective method. LPA is a statistical tool for grouping individuals (Asparouhov and Muthén, 2014). LPA and related methods, such as latent category analysis (LCA), are empirically derived techniques that identify latent subgroups in a population by maximizing intra-category homogeneity and inter-category heterogeneity between indicator variables. Compared to traditional cluster analysis, LPA does not require arbitrary assignment of the minimum distance between groups, nor does it require external discriminant analysis to check the relationship between exposure groups and results. Thus, LPA has advantages over other techniques, and the application of LPA to mental resilience can advance the theoretically driven literature. Since LPA is an individual-centered approach rather than a variable-centered approach, applying it to the resilience profile category of preschool left-behind children can help examine how demographic covariates predict profile membership and how problem behaviors differ across profiles.

At present, few studies on resilience have used LPA. Only three studies on children and adolescents have combined LPA and resilience. Duan used the Resilience Style Questionnaire (RSQ) to examine the resilience of poor children and adolescents aged 12 to 17 and obtained three types: (1) strength group, which had the highest scores on perseverance and optimistic approach to life; (2) risk group, which had the lowest scores; (3) common group, which had medium scores (Duan et al., 2020). Adolescents in the risk group reported more health problems and poorer peer relationships than others (Duan et al., 2020). Janousch adopted the Resilience Scale for Adolescents (READ) to analyze the resilience of adolescents aged 11 to 16 years in Germany, Greece, and Switzerland, and the results showed significant differences in the samples from different countries, which were divided into four types among the German and Greek adolescent groups: (1) non-resilient (high symptoms and low protective factors); (2) moderately resilient (moderate symptoms and moderate protective factors); (3) untroubled (low symptoms and high protective factors); (4) resilient (high symptoms and high protective factors). However, in the Swiss youth group, the participants were divided into three types: (1) non-resilient; (2) moderately resilient; (3) untroubled (Janousch et al., 2022). In addition, samples from different countries revealed differences in the proportions of individuals within each subgroup. The number of Greek adolescents in the resilient profile was almost twice that of German adolescents (Janousch et al., 2022). Yates did not use a special scale of resilience when analyzing the resilience of adolescents from nursing institutions. Instead, they used educational competence, occupational competence, civic engagement, relational competence, self-esteem, and depressive symptoms to represent resilience and identified four groups: (1) Maladapted: This group was characterized by adjustment difficulties in both external and internal domains of adaptation, with particularly notable difficulties in relationships; (2) Resilient: This group performed reasonably well in all areas; (3) Internally resilient: This group evidenced significant deficits in competence across multiple external adaptive domains but fared reasonably well in relationships and endorsed higher levels of self-esteem and lower levels of depressive symptoms relative to the other groups; (4) Externally resilient: Although this group appeared to be reasonably well-adjusted in the areas of education and civic engagement, they endorsed clinically significant levels of depressive symptoms (Yates and Grey, 2012). Adolescents in the maladapted profile group showed a higher incidence of problem behaviors. In contrast, adolescents in the resilient profile had fewer behavioral difficulties (Yates and Grey, 2012).

These studies preliminarily demonstrate the utility of LPA in examining resilience profiles and fill some gaps in the literature. Although Duan has researched resilience profiles and types of resilience (Duan et al., 2020; Janousch et al., 2022; Yates and Grey, 2012), it is unclear whether these findings can be generalized to younger age groups, especially preschool left-behind children. This population may develop distinct resilience profiles because early childhood is a sensitive period for attachment, emotion regulation, and social development. Prolonged parent–child separation, lack of stable family structure, and inadequate caregiving during these formative years can fundamentally alter how resilience manifests, making the patterns in preschool left-behind children likely to differ from those observed in older left-behind children or general child populations. Therefore, identifying specific resilience profiles in this vulnerable group carries important practical significance for promoting their mental health.

Resilience measurement tools can be used to screen individuals with different levels of resilience associated with health outcomes (Yu et al., 2011). At present, the measurement mainly focuses on adolescents; for example, the widely used Resilience Style Questionnaire (Duan et al., 2020) and the Resilience Scale for Adolescents (Janousch et al., 2022), which were designed and translated to assess the resilience of adolescents in different countries. Faced with a gap in tools to measure resilience in preschool children, LeBuffe developed a new measure, the Devereux Early Childhood Assessment for Preschoolers, Second Edition (DECA-P2), which was used to assess the resilience of preschool children. It is a behavioral rating scale used to assess child protective factors that are critical to social and emotional health and resilience. Specifically, it includes attachment/relationship, initiative, and self-regulation. The reliability and validity of this scale have been tested in a group of preschool children in China, and the results further confirmed the associations between the three protective factors of DECA-P2 and children’s emotional and behavioral problems (Zhang J. et al., 2021). The DECA-P2 is expected to have a screening function that separates at-risk individuals from others for further intervention. In this study, LPA will be used for analyzing its screening effect.

### 1.3 The current study

Given the importance of resilience to the mental health development of left-behind children, there is no published research on resilience profiles of preschool children. This study uses preschool left-behind children as the sample and applies LPA to the resilience indicators of preschool left-behind children in order to address important limitations in the existing literature. This study addresses the following issues: the lack of research on preschool children within the literature on resilience and the lack of individual-centered approaches rather than variable-centered studies on resilience. In addition, it is important to identify and protect low-resilience groups that are at higher risk; however, there is currently a lack of sociodemographic reference features to identify at-risk groups among preschool children. In addition, because problem behaviors play an important role in children’s physical and mental development, it is also necessary to explore the differences in various resilience profiles regarding these behaviors. Therefore, the purpose of this study is to build upon previous studies using LPA. Specifically, we aim to: (a) use DECA-P2 metrics related to attachment/relationship, initiative, and self-regulation to determine the potential profile types of resilience of preschool left-behind children; (b) explore how profile membership types differ based demographic characteristics. (c) examine whether significant differences exist in problem behaviors among children belonging to different profile types. The goal is to identify groups at higher risk to promote the healthy development of preschool left-behind children.

## 2 Materials and methods

### 2.1 Participants

A convenience sampling method was used to distribute online questionnaires to 1,078 parents of preschool left-behind children (ages 3–6) in nearly 30 kindergartens across the Zhejiang and Shandong provinces. The exclusion criteria for questionnaires were as follows: (1) the consistency rate of answers to reversed items or similar items exceeded 85%, indicating random or patterned responding; (2) more than 10% of the items were left unanswered; and (3) the questionnaire completion time was <5 min (which was determined to be too short for thoughtful completion based on a pilot test). After removing invalid questionnaires, 940 valid questionnaires were obtained, with a recovery rate of 87.1%. The questionnaires that met the exclusion criteria were deemed invalid and removed from the dataset (listwise deletion). For the remaining 940 valid questionnaires, missing data on individual scale items (less than 1%) were handled using Maximum Likelihood (ML) estimation during the analysis. There were 855 (91.0%) mothers, 50 (5.3%) fathers, and 35 (3.7%) other respondents. The mean age of participants was 5.07 years (SD = 0.80, range = 4.00–6.00). There were 484 boys (51.5%) and 456 girls (48.5%).

Data for this study were collected via the Internet. With the assistance of kindergartens, questionnaires were distributed through a WeChat group to parents of preschool children. Before completing each questionnaire, parents were asked about their willingness to participate. If a parent selected “I agree to participate in this survey,” indicating voluntary participation, the page for completing the questionnaire would be displayed. If parents selected “I do not want to participate in this survey,” indicating their lack of willingness to participate, the survey would end at this point. Following the ethical principle of voluntary participation, all participating parents and their children’s teachers signed informed consent forms. In accordance with the guiding principles of the Declaration of Helsinki, the research ethics committee of Guangzhou University approved the ethical review of this study (Guangzhou University Ethics [2024] No. 027).

### 2.2 Measures and data collection

#### 2.2.1 Personal information form

The Personal Information Form includes child’s age, sex, parents’ highest education level ranging from 1 (*primary school*) to 6 (*bachelor’s degree or above*), and annual family income level ranging from 1 (*less than 2,000 RMB*) to 9 (*more than 100,000 RMB*).

#### 2.2.2 Resilience

The Devereux Early Childhood Assessment for Preschoolers, Second Edition, (DECA-P2) was adopted to measure the resilience of preschool children (LeBuffe and Naglieri, 2013). DECA-P2 is a behavioral rating scale used to assess children’s protective factors critical to social and emotional health and resilience and to screen children for problem behaviors. DECA-P2 contains 38 items on a five-point Likert scale ranging from 0 (*never*) to 4 (*always*), with higher scores indicating higher levels of resilience in children. In this study, 27 items from the three protective dimensions of the scale were selected for measurement. The scale has proven to be reliable and valid in Chinese populations (Zhang J. et al., 2021). The total scale and each dimension in this study had Cronbach’s 𝛼 coefficients of 0.96, 0.88, 0.91, and 0.91.

#### 2.2.3 Problem behaviors

The Social Skills Improvement System—Rating Scales (SSIS-RS; parental version) Problem Behavior Subscale was used to understand the development of problem behaviors in children (Gresham and Elliott, 2008). The scale has 33 items and 5 dimensions, including externalizing problems, bullying problems, hyperactivity/inattention problems, internalizing problems, and autism spectrum problems. Externalizing problem behaviors include verbal or physical aggression, losing control of one’s temper, and arguing. Bullying problem behaviors include bullying others, forcing children to do things against their will, and intimidating them. Hyperactivity/inattention problems include being easily distracted or inattentive. Internalizing problem behaviors include restlessness, frequent sadness, and low mood. Autism spectrum problem behaviors include acting lonely and avoiding others. A 4-point Likert scale was used based on how often the child’s behaviors occur, ranging from 0 (*never*) to 3 (*almost always*). The higher the score, the more severe the child’s problem behaviors. The scale has proven to be reliable and valid in Chinese populations (Wang et al., 2021). The total scale and each dimension in this study had Cronbach’s 𝛼 coefficients of 0.96, 0.86, 0.84, 0.84, 0.85, and 0.80.

### 2.3 Data analysis

In this study, Mplus Version 7 was used for latent profile analysis (Muthén and Muthén, 2000), and the average score of the three dimensions of resilience was taken as the indicator in the model. The Robust Maximum Likelihood (MLR) estimator was used for parameter estimation, and Full Information Maximum Likelihood (FIML) was employed to handle missing data. To ensure model convergence and avoid local maxima, 500 random sets of starting values with 100 final-stage optimizations were specified for each model. Akaike Information Criterion (AIC), Bayesian Information Criterion (BIC), Adjusted BIC (aBIC), Lo–Mendell–Rubin test (LMRT), bootstrap likelihood ratio test (BLRT), entropy, and other fitting information indicators were selected to determine the most appropriate classification. As the number of profiles increased, we compared model fits until we obtained the best profile solution. The lower the AIC, BIC, and aBIC values, the better the model fitting (Muthén and Muthén, 2010). The *p*-values of LMRT and BLRT indicated that the k-profile model is significantly superior to the (k − 1)-profile model (Haridas et al., 2017). The entropy measure ranges from 0 to 1. When the entropy value is greater than 0.80, the classification accuracy is above 90% (Carragher et al., 2009).

After determining the optimal model, the Statistical Package for the Social Sciences (SPSS; IBM, Armonk, New York, USA) was used to examine the associations between profile membership, demographic characteristics, and problem behaviors. Multinomial logistic regression models were used as functions of demographic covariates to predict profile membership. This analysis compares the likelihood of membership in each profile relative to the reference profile (Asparouhov and Muthén, 2014). One-way analysis of variance was used to compare problem behaviors across profiles.

## 3 Results

The means, standard deviations, and bivariate correlations of the variables in this study are shown in Table 1.

**Table 1:** Means, standard deviations, and correlations among study variables.

Study variables	1	2	3	4	5	6	7	8	9
1 Attachment/Relationships									
2 Initiative	0.825^***^								
3 Self-regulation	0.814^***^	0.889^***^							
4 Externalizing	−0.233^***^	−0.269^***^	−0.334^***^						
5 Internalizing	−0.244^***^	−0.244^***^	−0.272^***^	0.759^***^					
6 Hyperactivity/Inattention	−0.198^***^	−0.271^***^	−0.334^***^	0.801^***^	0.736^***^				
7 Autism spectrum	−0.188^***^	−0.208^***^	−0.270^***^	0.760^***^	0.797^***^	0.808^***^			
8 Bullying	−0.250^***^	−0.247^***^	−0.294^***^	0.809^***^	0.744^***^	0.785^***^	0.796^***^		
9 Overall Problem Behaviors	−0.240^***^	−0.271^***^	−0.330^***^	0.902^***^	0.887^***^	0.920^***^	0.924^***^	0.903^***^	
Mean	23.06	20.77	20.52	7.65	8.89	11.86	11.34	7.09	46.82
SD	7.42	7.37	7.44	2.53	2.82	3.56	3.33	2.50	13.40

*p* < 0.001.

### 3.1 Latent profile analysis

As shown in Table 2, although AIC, BIC, and aBIC continued to decrease as the number of profiles increased, the reduction slowed down after the 4-profile model, indicating diminishing returns in fit improvement. While the 6-profile model also showed significant LMR and BLR tests, the LMR test for the 5-profile model was non-significant (*p* = 0.129), suggesting that the 4-profile solution was the last model to provide a significant improvement over the *k* − 1 solution in a stable manner.

**Table 2:** Model fit indices for 1- to 8-profile solutions based on three indices of resilience.

Profiles	AIC	BIC	aBIC	Entropy	BLRT (*p*-value)	LMR (*p*-value)	Proportion
1-Profile	19306.357	19335.432	19316.376	—	—	—	—
2-Profile	18141.122	18189.580	18157.821	0.807	0.000	<0.001	0.48/0.52
3-Profile	17317.743	17385.585	17341.122	0.880	0.000	<0.001	0.13/0.53/0.34
4-Profile	**16927.658**	**17014.884**	**16957.717**	**0.872**	**0.000**	**<0.001**	**0.38/0.34/0.18/0.10**
5-Profile	16702.314	16808.924	16739.053	0.890	0.000	0.129	0.02/0.12/0.36/0.33/0.17
6-Profile	16587.267	16713.260	16630.686	0.860	0.000	0.033	0.02/0.26/0.11/0.32/0.10/0.19
7-Profile	16528.289	16673.665	16578.387	0.847	0.000	0.134	0.08/0.02/0.24/0.09/0.29/0.10/0.17
8-Profile	16494.023	16658.783	16550.801	0.851	0.000	0.141	0.08/0.02/0.25/0.02%/0.09/0.27/0.17/0.10

AIC, Akaike information criterion; BIC, Bayesian information criterion; aBIC, adjusted Bayesian information criterion; BLRT, bootstrap likelihood ratio test. LMRT = Lo–Mendell–Rubin Adjusted test. The content of the bolded line is the final selected profile.

Furthermore, from the perspective of parsimony and class size, the 6-profile model generated a very small group (only 2% of the sample, *n* = 18), which may lead to low statistical power and poor representativeness. In contrast, the 4-profile model maintained adequate class sizes (all groups >10%) and provided the most theoretically meaningful and interpretable patterns of resilience. Therefore, considering both statistical indices and conceptual clarity, the 4-profile solution was selected as the optimal solution.

Figure 1 and Table 3 showed the standardized mean values of the resilience indices in the four profiles. The participants were divided into four profiles: (1) High-resilience group (profile 1, *n* = 164, 18% of the sample), scored highest on attachment/relationship, initiative, and self-regulation. (2) Medium-resilience group (profile 2, *n* = 324, 34% of the sample) scored at a moderate level. (3) Low-resilience group (profile 3, *n* = 355, 38% of the sample) had a low score. (4) Risk group (profile 4, *n* = 97, 10% of the sample) had the lowest score. The high-resilience group scored highest on attachment/relationships, initiative, and self-regulation; the medium-resilience group scored in the middle; and the low-resilience group scored lower, while the risk group scored the lowest.

**Figure 1:**
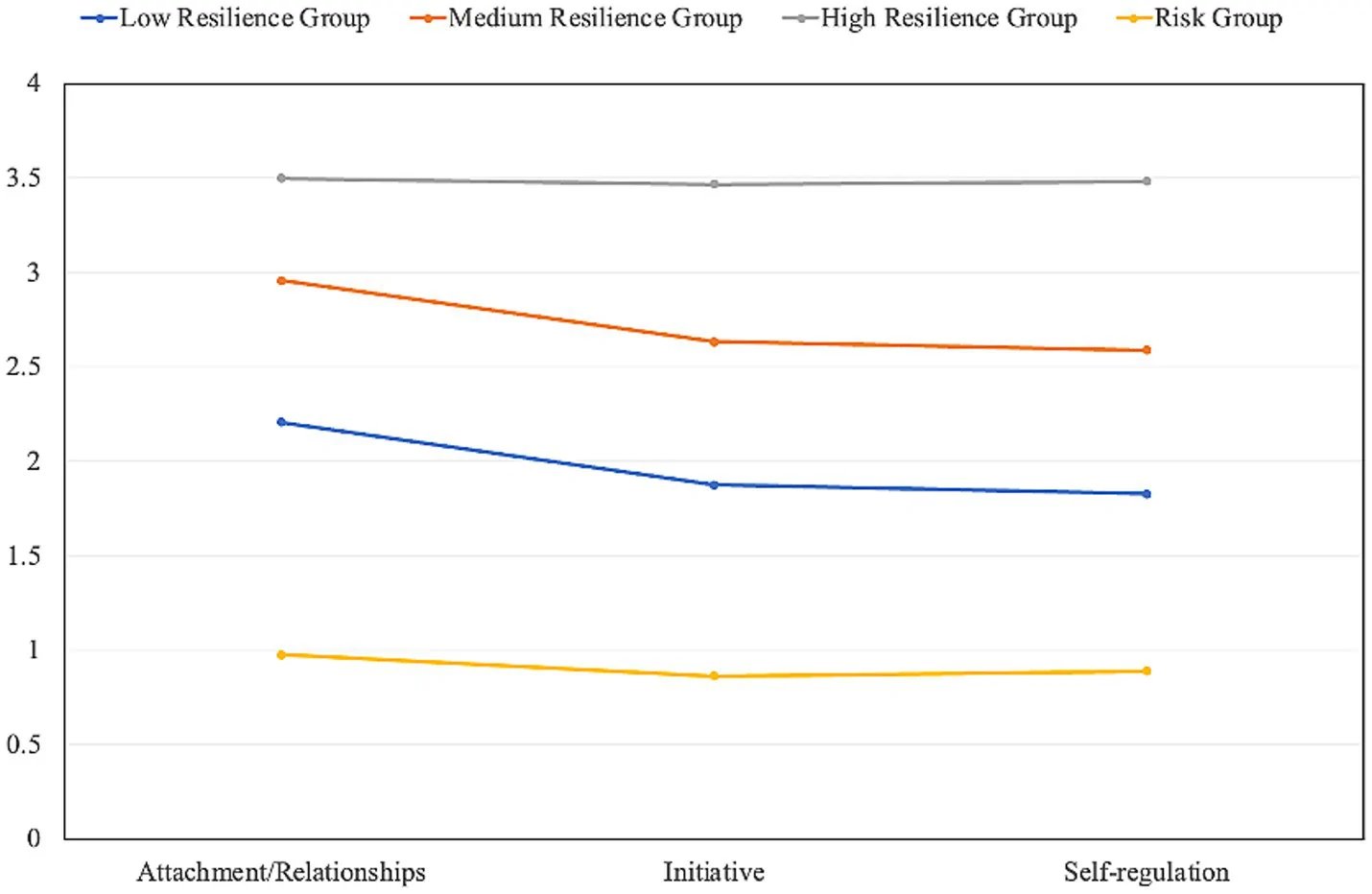
Standardized means of resilience indices across the four profiles.

**Table 3:** Mean and standard deviation of resilience in latent profile groups.

Resilience	Low resilience (*n* = 355, 37.7%)		Medium resilience (*n* = 324, 34.5%)		High resilience (*n* = 164, 17.5%)		Risk (*n* = 97, 10.3%)	
	M	SD	M	SD	M	SD	M	SD
Attachment/relationships	2.21	0.44	2.95	0.38	3.50	0.32	0.97	0.51
Initiative	1.87	0.32	2.63	0.31	3.47	0.36	0.86	0.42
Self-regulation	1.83	0.33	2.59	0.35	3.48	0.34	0.89	0.40

### 3.2 Child demographic variables associated with profile membership

Table 4 showed the differences in demographic covariates, including sex, age, parental education, and annual family income. The results of multinomial logistic regression analysis showed that boys were more likely to be classified as the low-resilience group and risk group, while girls were more likely to be classified as the high-resilience group. Compared with the high-resilience group, younger children were more likely to be classified as the medium-resilience group (that is, less likely to be classified as the high-resilience group than those in the medium-resilience group). Children whose parents had low levels of education were more likely to be classified as the low-resilience group and the risk group compared to the high-resilience group. Children from families with lower income levels were more likely to be classified as the risk group.

**Table 4:** Results of multinomial logistic regression for demographic covariates.

Demographic covariates	Low resilience			Medium resilience			Risk		
	*β*	SE	Odds ratio (95% CI)	*β*	SE	Odds ratio (95% CI)	*β*	SE	Odds ratio (95% CI)
Gender^a^	−0.39^*^	0.19	0.68 (0.46, 0.99)	−0.32	0.19	0.73 (0.50, 0.106)	−0.60^*^	0.27	0.55 (0.33, 0.93)
Age	−0.20	0.12	0.82 (0.65, 1.04)	−0.25^*^	0.12	0.78 (0.61, 0.99)	−0.11	0.17	0.90 (0.64, 1.25)
Parents’ education	−0.40^***^	0.09	0.67 (0.56, 0.80)	−0.13	0.09	0.88 (0.74, 0.1.04)	−0.48^***^	0.13	0.62 (0.49, 0.79)
Household income	−0.06	0.05	0.94 (0.85, 1.04)	0.02	0.05	1.01 (0.91–1.13)	−0.20^**^	0.06	0.82 (0.73, 0.93)

The high resilience profile served as the reference profile.

^*^p < 0.05, ^**^p < 0.01, ^***^p < 0.001.

a Gender was coded as 0 = boys and 1 = girls.

### 3.3 Latent profile and problem behaviors of left-behind children

Analysis of variance was used to examine differences between groups in problem behaviors among the low resilience, medium resilience, high resilience, and risk groups. The Bonferroni method was used for statistical analysis. The total score of problem behaviors and the mean and standard deviation of each dimension in each group are shown in Table 5. As shown in Table 5, an one-way analysis of variance (ANOVA) revealed significant differences across the four profiles for all problem behavior variables (*p* < 0.001). The effect sizes, as measured by partial eta squared (*η*_p_^2^), ranged from 0.09 to 0.16, indicating medium to large effects (Cohen, 1988).

**Table 5:** Estimated differences in problem behavior variables across latent profile groups.

Variables	Low resilience		Medium resilience		High resilience		Risk		*η_p_* ^2^
	M	SD	M	SD	M	SD	M	SD	
Externalizing	7.75^a^	2.18	7.55^a^	2.18	6.47^b^	2.66	9.74^c^	3.42	0.12
Internalizing	9.24^a^	2.61	8.52^b^	2.40	7.82^c^	3.12	10.69^d^	3.12	0.10
Hyperactivity/inattention	12.30^a^	3.11	11.54^b^	3.10	10.15^c^	3.99	14.32^d^	4.17	0.13
Autism spectrum	11.68^a^	3.02	11.15^b^	3.06	10.04^c^	3.91	13.34^d^	3.49	0.09
Bullying	7.23^a^	2.27	6.87^a^	2.16	6.27^b^	2.76	9.30^c^	3.09	0.11
Overall problem behaviors	48.20^a^	11.60	45.64^b^	11.33	40.75^c^	15.48	57.39^d^	16.61	0.16

Means with the same superscript are not significantly different at the *p* < 0.05 level.

The risk group scored the highest for in externalizing (M = 9.74, SD = 3.42), internalizing (M = 10.69, SD = 3.12), hyperactivity/inattention (M = 14.32, SD = 4.17), autism spectrum (M = 13.34, SD = 3.49), bullying (M = 9.30, SD = 3.09), and overall problem behaviors (M = 57.39, SD = 16.61) scored the highest, while the high-resilience group in externalizing (M = 6.47, SD = 2.66), internalizing (M = 7.82, SD = 3.12), hyperactivity/inattention (M = 10.15, SD = 3.99), autism spectrum (M = 6.27, SD = 2.76), and overall problem behaviors (M = 40.75, SD = 15.48) scored the lowest. By comparison, the results showed that there were significant differences in the scores of internalizing, hyperactivity/inattention, autism spectrum, and problem behaviors between the low-resilience group, the medium-resilience group, the high-resilience group, and the risk group. In terms of externalizing and bullying, there was no significant difference between the low-resilience group and the medium-resilience group, but there were significant pairwise differences between the other groups.

## 4 Discussion

In this study, the LPA method was used to analyze the resilience of preschool left-behind children based on three factors of resilience and to explore the relationship between their demographic characteristics, problem behaviors, and resilience. The results showed that the resilience of preschool left-behind children could be divided into four profiles.

### 4.1 Heterogeneity of resilience among preschool left-behind children

To the best of our knowledge, this study is the first to identify the resilience profiles of preschool left-behind children in China based on the DECA-P2. Through LPA, four homogeneous profiles were found: the high-resilience group (17.5% of the sample), which had the highest scores in attachment/relationships, initiative, and self-regulation. The medium-resilience group (34.5% of the sample) had an overall score at the medium level, among which attachment/relationships was higher than initiative and self-regulation levels. The low-resilience group (37.7% of the sample) had a low overall score, among which attachment/relationships was higher than initiative and self-regulation levels. While the risk group (10.3% of the sample) had the lowest scores on attachment/relationships, initiative, and self-regulation. According to the ecological systems theory (Bronfenbrenner, 1979), these distinct resilience profiles are not merely individual traits but outcomes of the dynamic interaction between children and their multi-layered environments. The distribution of these four groups suggests that preschool left-behind children occupy different ecological niches. Overall, the medium- and low-resilience groups comprised the majority of children, exhibiting moderate and low resilience levels in the majority of areas. In other words, the majority of left-behind children did not demonstrate high levels of resilience. This was consistent with the findings of a previous study on resilience in poor children and adolescents aged 12 to 17, which found that the majority of the sample consisted of subgroups characterized by moderate and low levels of resilience (Duan et al., 2020).

Compared to previous studies, this study found a new subgroup—the risk group. Left-behind children in the risk group scored very low in all areas of resilience (the mean of all areas of resilience was less than 1). This may be because the majority of participants in previous studies were ordinary teenagers. In contrast, left-behind children’s early experiences have changed their family and social environments, disrupted their physical and mental health, and hindered their overall development. As a result, their overall level of resilience is low (Ding et al., 2022; Wu et al., 2017). Left-behind children, as a special group, face disadvantageous situations. While some left-behind children with high resilience levels can still achieve healthy growth, others may be seriously negatively affected by the environment and are more likely to collapse in the face of difficulties (van Breda, 2017). Therefore, it is urgent to screen and implement intervention measures for high-risk left-behind children.

### 4.2 Differences in problem behaviors of preschool left-behind children of different resilience profiles

In this study, the risk group with the lowest level of resilience reported more externalizing problems, internalizing problems, hyperactivity/inattention problems, autism spectrum problems, and bullying problems than the other three groups. This is consistent with previous studies, in which resilience is a negative predictor of emotional and behavioral problems (Ai and Hu, 2016; Ding et al., 2022). These results support the conclusion that resilience may play a protective role in the problem behaviors of preschool left-behind children (Ding et al., 2022). In other words, children with high levels of resilience adapt positively to the adversity of being left behind for a long time and quickly return to baseline levels (Olick, 2016), so the frequency of problem behaviors is lower. Therefore, resilience may be a means to improve the problem behaviors of children left behind in long-term adversity. Preschool left-behind children can benefit from interventions aimed at improving resilience, especially in terms of improving problem behaviors.

### 4.3 Demographic characteristics of preschool left-behind children with different resilience profiles

The results of this study showed that left-behind boys were more likely to be classified as the low-resilience group and risk group, while girls were more likely to be classified as the high-resilience group. This is consistent with previous studies showing that left-behind girls show higher levels of resilience than left-behind boys (Chen et al., 2021; Sun and Stewart, 2007). The results can be explained by relationship theory, which suggests that girls tend to explore who they are in relationships with others rather than in isolation (Miller, 1986) and seek empathic connections in all major relationships. A fundamental aspect of girls’ psychological growth is to see themselves as a relational entity, and these relationships lead to positive outcomes such as better communication, positive feelings about the self, and a strong sense of identity (Patterson, 2001), as well as the use of more emotion-centered strategies such as communication, empathy, and help-seeking strategies. Therefore, left-behind girls, when faced with the adversity of staying behind for a long time, will have a stronger ability to bear and a higher level of resilience.

This study found that, compared with the medium-resilience group, younger left-behind children were more likely to be classified as the high-resilience group. This is consistent with previous research results (Sun and Stewart, 2007). This may be because left-behind children’s beliefs in the importance and effectiveness of support from adults in their families, support from adults in their communities, and peer support at school increase as they age. In addition, their competency beliefs about success, such as problem-solving, also increased over time. Therefore, in the face of long-term adversity, older left-behind children are more confident that they can effectively overcome difficulties and have a higher level of resilience.

The study also found that left-behind children whose parents had low levels of education were more likely to be classified as the low-resilience group and higher risk compared to the high-resilience group. It also supports the assertion that low parental education is a risk factor for children’s resilience (De Feyter et al., 2020; Li and Yeung, 2019; Perez et al., 2009). From the perspective of ecological systems theory (Bronfenbrenner, 1979), parental education serves as a critical distal factor that shapes the quality of the family microsystem. Highly educated parents may possess greater cultural capital, which allows them to provide more cognitively stimulating environments and maintain more effective proximal processes—the primary engines of development—even across geographical distances. This may be because more educated parents also have higher expectations for their children’s development. Children who believe their parents have high expectations of them tend to invest more in them (Martin and Hau, 2010). Children tend to see qualities such as their strength, resilience, and independence as a responsibility to their parents (Li and Yeung, 2019). Therefore, in the face of long-term adversity, left-behind children whose parents have a higher level of education will devote more energy to overcoming difficulties and have a higher level of resilience.

As shown in previous studies (Smith-Adcock et al., 2019), left-behind children with lower family income levels are more likely to be classified as the risk group. According to the Family Stress Model within the ecological framework, family income level is an exosystemic factor that profoundly influences the stability of the child’s immediate environment. Economic hardship often creates a “spillover effect,” where financial strain at the macrosystem level (poverty) infiltrates the microsystem, compromising the quality of caregiving (Conger and Donnellan, 2007). This may be because families at the lower end of the income scale tend to focus on improving their finances rather than family relationships and parenting. Children from families with higher income levels were more likely to be classified as the high-resilience group, possibly because families with higher income levels may have a better balance between work and childcare (Dong et al., 2021). These findings illustrate how multiple contextual factors—education and income—jointly shape resilience profiles: while education provides the psychological resources for resilience, income provides the material stability necessary to sustain those resources. For children in the risk group, the convergence of low parental education and low family income creates an ecological disadvantage that severely limits their ability to develop self-regulation and secure attachment. Therefore, left-behind children with low family income generally receive a lower level of support and care from their families and have less confidence in the face of adversity, as well as a lower level of resilience.

## 5 Conclusion

In summary, this study identifies the heterogeneity of resilience among preschool left-behind children by distinguishing four distinct profiles: high resilience, medium resilience, low resilience, and risk groups. The results highlight that these resilience subtypes are significantly associated with specific demographic characteristics and varying levels of problem behaviors. While these findings provide a foundation for understanding the diverse needs of this population, it is important to note that the current study did not directly test the effectiveness of specific interventions. Therefore, the practical implications for individualized support should be interpreted with caution. Future research using longitudinal designs is essential to validate the stability of these latent profiles over time and to determine their long-term developmental trajectories. Additionally, multi-informant studies—incorporating data from multiple sources such as parents, teachers, and caregivers—are needed to confirm the predictive utility of these profiles. Such research will provide a more robust and comprehensive basis for developing evidence-based interventions tailored to the unique resilience patterns of left-behind children.

### 5.1 Limitations

Nevertheless, the current study suffers from three limitations. First, the participants in this study were mainly from two provinces in China—the Zhejiang and Shandong provinces on the southeast coast of China. Compared with the provinces in northwest China, these provinces have better economic and medical development. Therefore, the results cannot be fully generalized to other regions. Future studies can explore the differences in latent categories and influencing factors of resilience of preschool left-behind children through the comparison of different countries and regions. Second, this study adopts a cross-sectional design, which cannot discuss the dynamic changes in different profile groups from a longitudinal perspective. Future studies can adopt a longitudinal research method of tracking, such as the establishment of a latent profile growth model, to explore the longitudinal changes in the resilience of preschool left-behind children. Finally, the data in this study came from the reports of the children’s parents, and a single data source may have some errors. A multi-provider approach may be adopted in the future.

## Data Availability

The raw data supporting the conclusions of this article will be made available by the authors, without undue reservation.
